# Evaluation of Fermented Soybean Meal to Replace a Portion Fish Meal on Growth Performance, Antioxidant Capacity, Immunity, and mTOR Signaling Pathway of Coho Salmon (*Oncorhynchus kisutch*)

**DOI:** 10.1155/2023/2558173

**Published:** 2023-07-25

**Authors:** Qin Zhang, Mengjie Guo, Fanghui Li, Meilan Qin, Qiuyue Yang, Hairui Yu, Jian Xu, Yongqiang Liu, Tong Tong

**Affiliations:** ^1^Guangxi Key Laboratory for Polysaccharide Materials and Modifications, School of Marine Sciences and Biotechnology, Guangxi Minzu University, 158 University Road, Nanning 530008, China; ^2^Key Laboratory of Biochemistry and Molecular Biology in Universities of Shandong (Weifang University), Weifang Key Laboratory of Coho Salmon Culturing Facility Engineering, Institute of Modern Facility Fisheries, Weifang University, Weifang 261061, China

## Abstract

In this study, we evaluated the effects of fermented soybean meal (FSBM) or/and unfermented SBM replacing a portion of fish meal (FM) on the growth performance, antioxidant capacity, immunity, and mechanistic target of rapamycin (mTOR) signaling pathway of juvenile coho salmon (*Oncorhynchus kisutch*). Four groups of juvenile coho salmon (initial weight 152.23 ± 3.21 g) in triplicate were fed for 12 weeks on four different iso-nitrogen and iso-lipid experimental diets: G0 diet (28% FM protein, control group), G1 diet (18% FM protein and 10% SBM protein), G2 diet (18% FM protein, 5% SBM protein, and 5% FSBM protein), and G3 diet (18% FM protein and 10% FSBM protein). The main results were compared with the G0 diet; the weight gain rate, specific growth rate, and condition factor of juveniles in G3 were increased significantly (*p* < 0.05). The content of muscle crude protein, the total protein, glucose, albumin, total cholesterol in serum, and the total antioxidant capacity in the liver of juveniles in G3 was increased significantly (*p* < 0.05). The activities of pepsin, trypsin, *α*-amylase, and lipase in the intestine, the superoxide dismutase, catalase, and alkaline phosphatase in the liver of juveniles in G3 were increased significantly (*p* < 0.05). The expression levels of phosphatidylinositide 3-kinases, serine/threonine kinase, mTOR, and ribosomal protein S6 kinase 1 genes in the liver of juveniles in G3 were upregulated significantly (*p* < 0.05). The feed coefficient ratio, viscerosomatic index, the contents of muscle moisture, and malondialdehyde in the liver of juveniles in G3 were decreased significantly (*p* < 0.05). The expression levels of tumor necrosis factor *α*, interleukin 1*β*, and interleukin 6 genes in the liver of juveniles in G3 were downregulated significantly (*p* < 0.05). However, there was no significant effect (*p* > 0.05) on the survival rate, food intake, and muscle crude lipid and ash of juveniles among the experimental groups. In conclusion, FSBM to replace a portion FM had a positive effect on the growth performance, protein deposition, antioxidant enzyme activity, digestive enzyme activity, protein synthesis, and immune-related genes of juvenile coho salmon.

## 1. Introduction

As a result of its high protein and fat content, fish meals (FMs) are frequently used in diets of carnivorous fish [1]. More than 70% of global aquaculture production depends on FM, and diets containing higher levels of FM lead to better fish growth and food conversion rate [2]. In recent decades, the excessive development of fish wild populations has limited the availability and price of FM, a major protein source in aquaculture feedstuff [3]. To reduce feed costs and promote the sustainable development of fish farming, it is important to find alternative sources of FM in fish feed [4].

Aquaculture relies heavily on plant protein sources for continuous growth and intensification [5], such as soybean meal (SBM), corn gluten meal, rapeseed, cottonseed, and wheat protein concentrate [6]. In recent years, because of its high protein content (about 43%–48%), reasonable amino acid composition, wide source, and low price, SBM protein has become a candidate protein source to replace FM protein in aquatic feed [7, 8]. However, SBM contains a variety of antinutritional factors, such as soybean globulin, trypsin inhibitor, soybean lectin, and antivitamin [9–11], which hinder the efficient utilization of plant protein nutrition and limit the digestion and absorption of aquaculture animals, resulting in decreased growth performance [12, 13].

Nowadays, fermentation technology plays a significant role in feed production [14]. After microorganism fermentation, huge amounts of antinutritional factors are degraded [15]. Soybean protein is hydrolyzed into small molecular amino acids, peptides, and water-soluble compounds, producing some unknown bioactive substances, thereby improving the availability and palatability of feed nutrients [16]. The study indicates that fermentation of SBM under appropriate conditions is beneficial for preventing various physiological abnormalities that occur in rainbow trout fed SBM, and fermented SBM (FSBM) is a promising ingredient as the main protein source in a non-FM diet for rainbow trout (*Oncorhynchus mykiss*) [17]. Tsai et al. [18] fed an FM or SBM diet to Atlantic Salmon (*Salmo salar* L.), and the study has shown that Atlantic Salmon were sensitive to the inclusion of SBM in the diet, which can result in inflammation in the distal intestine. Nimalan et al. [19] found the protein components of fermented SBM feed could improve the digestion and absorption of feed and reduce the lamina propria width, which probably indicates the prevention of enteritis in Atlantic Salmon. Studies on aquatic animals have shown that the protein components of fermented SBM feed are more easily absorbed and utilized than those of unfermented SBM feed [20, 21].

Coho salmon (*Oncorhynchus kisutch*) has gradually become a vital choice for salmon farming because of its polyunsaturated fatty acids, vitamins and minerals, delicious taste, and high economic value [22–24]. FM is considered by far the most advantageous protein source for coho salmon, and 0.7 kg of marine protein was used to produce 1 kg of salmon protein, so the farmed salmon is thus a net producer of marine protein [25]. However, there aren't many reports on coho salmon fed FSBM instead of FM. The substitution of FM for FSBM was studied in coho salmon to integrate an assessment of its effects. *Bacillus pumilus* has been widely used in aquaculture research on account of its capacity to produce amylase and cellulase, extracellular protease, and antimicrobial peptide and has strong bacteriostatic ability. The protease produced by the metabolism of *B. pumilus* can help the fish digest the macromolecular protein in the feed and enhance the immunity of the fish [26]. The selection of *B. pumilus* as a fermentation strain, through the metabolism of the strain to produce antibiotics, not only can reduce the dependance of animals on antibiotics, promote growth but also prevent diseases and can be used as a substitute for antibiotics. In addition, *B. pumilus* has the characteristics of simple nutritional requirements, rapid reproduction, rapid metabolism, strong vitality, high temperature and high-pressure resistance, acid and alkali resistance, antioxidant, high oxygen and low oxygen resistance, and has a strong survival ability in the gut, and is very suitable for solid-state fermentation strains [27]. Therefore, the experiment aims at evaluating the effects of *B. pumilus* FSBM or/and unfermented SBMs replacing a portion of FM on the growth performance, antioxidant, immunity, and mechanistic target of rapamycin (mTOR) signaling pathway of juvenile coho salmon. The use of FSBM in coho salmon feed fills the knowledge gap and provides a theoretical basis for the development and optimization of coho salmon feed and commercial salmon culture.

## 2. Materials and Methods

### 2.1. Experimental Diets

The diet formula and approximate composition are shown in Table 1. Based on previous studies, protein from 10% FSBM could be substituted for FM protein in aquaculture animals [28, 29]. Therefore, a series of four different diets were designed for juvenile coho salmon, including an iso-nitrogenous diet (about 43% dietary protein) and an iso-lipid diet (about 15% dietary lipid), the control group (G0) contained 28% FM protein. In G1, G2, and G3 groups, 18% of the protein was derived from FM protein, in addition. 10% of the G1 group from unfermented SBM protein, 10% of the G3 group from FSBM protein, and the G2 group was composed of 5% unfermented SBM protein and 5% SBM protein.

*B. pumilus* was isolated from mangrove roots soil of Maowei Sea at 21°81′66′′N, 108°58′46′′E in Qinzhou, China. The fermentation parameters were optimized with the yield of crude protein as a reference [30]. A preliminary fermentation experiment in our laboratory determined the fermentation conditions. The brief steps of SBM fermentation were: first, the SBM and sterile water were mixed at the weight ratio of 1 : 1.2 and sterilized at 121°C for 30 min; second, after cooling to 25–30°C, 8% *B. pumilus* (v/m) was inoculated and fermented for 36 hr at 37°C; third, the mixture was dried at 30°C until the moisture content was below 100 g/kg. The nutritional composition and anti-nutritional factor content of SBM and FSBM were determined by Shanghai Panrui Technology Co., LTD (Shanghai, China), shown in Table 2.

All ingredients were ground to below 60 mesh and combined in a roller mixer for 15 min. The distilled water of equal weight was added to the fine powder to make a stiff dough, and a single screw extruder (Delun, Jinan Delun Machinery Equipment Co., LTD, Jinan, China) was used to obtain the feed particles with a diameter of 2.0 × 3.0 mm^2^. Then the feed particles were dried in the airflow at 30°C until the water content was less than 100 g/kg. The dried feed particles were put into the sealing pockets and stored at −20°C until for use.

### 2.2. Experimental Fish and Culture

A hatchery in Benxi Rainbow Trout Breeding Farm, Liaoning, China, provided juvenile coho salmon, and outdoor breeding experiments were conducted at the rainbow trout farm in Nanfen, Liaoning, China. All animal experiments were conducted in accordance with the guidelines of Guangxi Minzu University, Nanning, China, and this research does not contain any studies with human participants (approval number: GXUN 2021-006).

Before the experiment, the juveniles were fed with the G0 diet and acclimated in the net cages (1 × 1 × 0.5 m^3^) with water temperatures between 10 and 18°C, Sewage inflow ≥100 L/s, water surface current velocity ≥2 cm/s, dissolution oxygen ≥6.0 mg/L, pH 7.5–8.0, and natural light for 2 weeks.

After being acclimatized for 2 weeks, for the formal experiment, 390 fish (initial body weight 152.23 ± 3.21 g) were selected, 30 of which were taken as the initial samples. We divided the remaining 360 fish into four groups in triplicate, resulting in 12 net cages with 30 fish per cage (1 × 1 × 0.5 m^3^). As described in acclimatization progress, juvenile coho salmon were cultured in the same aquaculture system. The water was changed one-third a day. The juvenile coho salmon were artificially fed three times (8:00, 12:00, and 16:00) daily until they reached satiation. The juveniles were fed one of the four diets abovementioned for 12 weeks (Table 1).

### 2.3. Sampling Procedures

After 24 hr of starvation, juvenile coho salmon were separately sampled at 0 day and 12 weeks. A 30 mg/L dose of methanesulfonic acid tricaine (MS-222, Adamas Reagent, China) was used to anesthetize all samples. Then the body length and body weight of juvenile coho salmon were individually measured. Twenty of the juveniles were dissected for liver samples at 0 day, and another 10 were taken for whole fish samples. At the end of 12 weeks, six fish were randomly selected from each cage as samples, of which three were serum, liver, muscle, stomach, and intestine samples, and the remaining three were whole fish samples. Blood was taken from the caudal vein of coho salmon with a sterile syringe and transferred to a 2 mL sterile enzyme-free centrifuge tube and placed at 4°C for 2 hr. Then the blood samples were centrifuged at 3,500 *g* and 4°C for 15 min, and the supernatant was serum. No anticoagulant was utilized to obtain a serum sample. The liver and visceral mass were removed, weighed, and collected in a 20 mL centrifuge tube, respectively. All the samples were stored at −80°C before index determination.

### 2.4. Calculations and Analytical Methods

#### 2.4.1. Growth Performance

Survival rate (SR), weight gain rate (WGR), specific growth rate (SGR), feed coefficient ratio (FCR), condition factor (CF), hepatosomatic index (HSI), viscerosomatic index (VSI), and food intake (FI) were calculated using the following formula:

(1) $$\begin{array}{c} \text{SR}\;\left(\%\right)=100\times \frac{\text{final amount of fish}}{\text{initial amount of fish}}, \end{array}$$

(2) $$\begin{array}{c} \text{WGR }\left(\%\right)=100\times \frac{\text{final body weight }\left(g\right)-\text{initial body weight }\left(g\right)}{\text{initial body weight }\left(g\right)}, \end{array}$$

(3) $$\begin{array}{c} \text{SGR}\left(\%/\mathrm{day}\right)=100\times \frac{\left[\ln\;\left(\text{final body weight}\right)\;\left(g\right)\right]-\left[\ln\;\left(\text{initial body weight}\right)\;\left(g\right)\right]}{\text{days}}, \end{array}$$

(4) $$\begin{array}{c} \text{FCR}=\frac{\text{total feed intake }\left(g\right)}{\text{final body weight }\left(g\right)-\text{initial body weight }\left(g\right)}, \end{array}$$

(5) $$\begin{array}{c} \mathrm{CF}\;\left(\%\right)=100\times \frac{\text{body weight}\left(g\right)}{{\left[\text{body length }\left(\mathrm{cm}\right)\right]}^{3}}, \end{array}$$

(6) $$\begin{array}{c} \mathrm{HSI}\;\left(\%\right)=100\times \frac{\text{liver weight }\left(g\right)}{\text{body weight }\left(g\right)}, \end{array}$$

(7) $$\begin{array}{c} \mathrm{VSI}\;\left(\%\right)=100\times \frac{\text{viscera weight }\left(g\right)}{\text{body weight }\left(g\right)}, \end{array}$$

(8) $$\begin{array}{c} \mathrm{FI}=\frac{\text{total feed intake }\left(g\right)}{\text{days}}. \end{array}$$

#### 2.4.2. Evaluating the Approximate Composition of Feed and Muscle

Fish feed and muscle samples were examined for crude protein using the Kjeldahl apparatus (nitrogen 6.25) (HGK-55, Shanghai Heguan Instrument, Shanghai, China), crude lipid using ether extraction, ash using a muffle furnace heated to 550°C for 10 hr, and moisture using a 105°C oven [31].

#### 2.4.3. Serum Biochemical, Antioxidant, and Digestive Indexes Analysis

Using commercial reagent kits produced by the Nanjing Jiancheng Bioengineering Institute in Nanjing, China, all indices were derived. The glucose (GLU) oxidase technique was used to measure the GLU content at 505 nm. The cholesterol oxidase (COD-PAP) technique was used to measure the total cholesterol (T-CHO) level at 500 nm. The Coomassie brilliant blue technique was used to measure the total protein (TP) concentration at 595 nm. The bromocresol green technique was used to assess the albumin (ALB) level at 630 nm. The alkaline phosphate (AKP) activity was determined at 520 nm by the potassium ferricyanide oxidation method.

The samples of liver, stomach, and intestine were homogenized with 0.9% normal saline at 4°C and then centrifuged at 3,000 *g* for 10 min at the same temperature. For biochemical examination, the supernatant was collected. A colorimetric approach was used to measure the liver's total antioxidant capacity (T-AOC) at 520 nm. Using the ammonium molybdate methodology, the liver's catalase (CAT) activity was measured at 405 nm. Using the xanthine oxidase method, the liver's superoxide dismutase (SOD) activity was assessed at 550 nm. The thiobarbituric acid (TBA) method at 532 nm was used to assess the amount of malondialdehyde (MDA) in the serum and liver. The activity of pepsin in the stomach was measured at 660 nm by the phenol reduction method. The activity of trypsin in the intestine was measured at 253 nm according to the hydrolysis ability of the enzyme itself. The activity of *α*-amylase in the intestine was measured at 660 nm by iodine colorimetry. The activity of lipase in the intestine was determined at 580 nm by the methylic resorufin substrate method. All the above indexes were measured using a microplate reader (Tecan Spark10M, Salzburg, Austria).

#### 2.4.4. Real-Time Quantitative Polymerase Chain Reaction

*mTOR*, ribosomal protein S6 kinase 1 (*S6K1*), serine/threonine kinase (*AKT*), phosphatidylinositide 3-kinases (*PI3K*), tumor necrosis factor *α* (*TNF-α*), interleukin 1*β* (*IL-1β*), and interleukin 6 (*IL-6*) gene expression levels were determined using real-time quantitative polymerase chain reaction (RT-qPCR). Utilizing Thermo Fisher Scientific's Steady Pure Universal RNA Extraction Kit, total RNA from the liver was extracted, and its integrity was assessed by electrophoresis on 1.2% agarose gel and quantified by Nano Drop® 2000 spectrophotometer. Using the Evo M-MLV reverse transcription kit reversed transcription of RNA into cDNA. PCR conditions were set at 50°C for 30 min, 95°C for 5 min, and 5°C for 5 min. All kits were produced by Accurate Biology Biotechnology Engineering Ltd., Changsha, China.

The genomic sequences of coho salmon for *mTOR*, *S6K1*, *AKT*, *PI3K*, *TNF-α*, *IL-1β*, *IL-6*, and *β-actin* were obtained from the NCBI database. *β-actin* was selected as the internal reference gene. As stated in Table 3, the forward and reverse primers were created using Primer Premier 5.0 from Premier, Inc. and synthesized by Sangon Biotech (Shanghai) Co., Ltd.

The RT-qPCR was carried out using an SYBR Green Pro Taq HS qPCR kit from Accurate Biology Biotechnology Engineering Ltd., Changsha, China, and a LightCycler® 96 RT-qPCR equipment from Roche, Switzerland. The following RT-qPCR conditions were used, with a reaction volume of 20 *μ*L: 50°C for 30 min, 95°C for 5 min, and 5°C for 5 min. The *mTOR*, *S6K1*, *AKT*, *PI3K*, *TNF-α*, *IL-1β*, and *IL-6* genes' relative expression were determined using the 2^−*ΔΔCt*^ technique [32].

### 2.5. Statistical Analyses

The analyses were carried out using SPSS version 25.0 (Chicago, IL, USA), and all data were presented as means standard deviations (means ± SD). Normal distribution, two-way analysis of variance (ANOVA), and one-way ANOVA were employed for data analysis. Tukey's multiple range test was used to compare each other. It is significantly different when *p* < 0.05.

## 3. Results

### 3.1. Effect of Fermented Soybean Meal on the Growth Performance

The final weight, WGR, SGR, and CF of G3, as well as the HSI, VSI, and FCR of G1 and G2, were all significantly greater (*p* < 0.001) than those of the control group (G0). However, the final body weight, WGR, SGR, and CF of G1 and G2, and HSI, VSI, and FCR of G3 were reduced significantly (*p* < 0.001). There were no appreciable variations in the SR (*p* = 0.728) and FI (*p* = 0.974) of the juveniles among all groups, as shown in Table 4.

### 3.2. Effect of Fermented Soybean Meal on the Muscle Composition

The muscle moisture (*p* < 0.001) and crude protein (*p* < 0.001) of the G3 group were considerably higher compared to the control group (G0), while the muscle crude protein (*p* < 0.001) of the G1 group was significantly lower. The muscle moisture (*p* = 0.228) between G1, G2, and G0, as well as the muscle crude protein (*p* = 0.007) between G2 and G0, did not differ significantly. There were no significant differences in the muscle crude lipid (*p* = 0.959) and ash (*p* = 0.532) of the juveniles among all groups, as shown in Table 5.

### 3.3. Effect of Fermented Soybean Meal on the Serum Biochemical Indexes

As shown in Table 6, although the serum levels of the G1 and G2 groups were markedly lower (*p* < 0.001) than those of the control group (G0), the G3 group had markedly higher (*p* < 0.001) serum levels of GLU, T-CHO, TP, ALB, and AKP.

### 3.4. Effect of Fermented Soybean Meal on the Activities of Digestive Enzyme

Pepsin, trypsin, lipase, and *α*-amylase levels in the G3 group were substantially greater (*p* < 0.001) than in the control group (G0), although they were significantly lower (*p* < 0.001) in the G1 and G2 groups, as shown in Table 7.

### 3.5. Effect of Fermented Soybean Meal on the Activities of Antioxidant Enzyme

There was a significant increase (*p* < 0.001) in the content of T-AOC, CAT, and SOD activities in the liver of the G3 group compared to the control group (G0) and an increase in MDA content in the liver of the G1 group compared with the G2 group. Nevertheless, T-AOC, as well as CAT and SOD activities and MDA content, were significantly lower (*p* < 0.001) in G1 and G2. as shown in Table 8.

### 3.6. Effect of Fermented Soybean Meal on the Relative Expressions of Immunity and mTOR Signaling Pathway Gene

**Figure 1:**
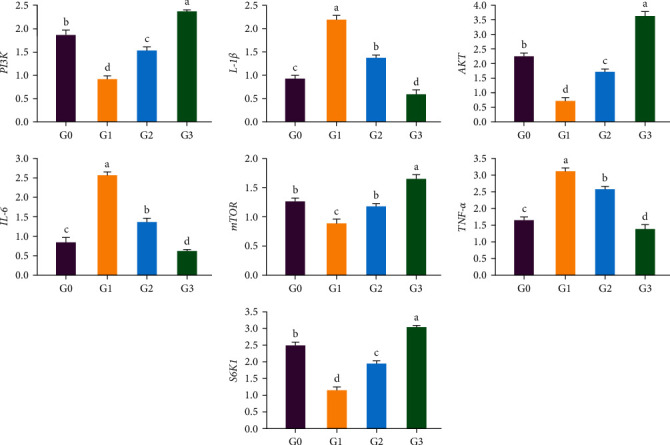
Effect of replacing a portion fish meal with unfermented and/or fermented soybean meal on the relative expression of phosphatidylinositide 3-kinases (*PI3K*), serine/threonine kinase (*AKT*), mechanistic target of rapamycin (*mTOR*), ribosomal protein S6 kinase 1 (*S6K1*), interleukin 1*β* (*IL-1β*), interleukin 6 (*IL-6*), and tumor necrosis factor *α* (*TNF-α*) gene in the liver of juvenile coho salmon. *β-Actin* was the internal reference gene. All above data are mean ± SD (*n* = 3 × 3), which means three parallel groups for each experimental group and three fish samples for each parallel group. G0 is the control group. G1 is the SBM group. G2 is the SBM and FSBM group. G3 is the FSBM group. Different superscript letters in the same row indicate significant differences among the data (*p* < 0.05).

The relative expressions of the *IL-1β*, *IL-6*, and *TNF-α* genes in the liver of the G3 group were substantially lower (*p* < 0.001) than those of the control group (G0), whereas those of the G1 and G2 groups were significantly greater (*p* < 0.001), as shown in Figure 1.

The relative expressions of *PI3K*, *AKT*, *mTOR*, and *S6K1* in the liver of the G3 group were considerably greater (*p* < 0.001) than those of the control group (G0), but those of *PI3K*, *AKT*, *mTOR*, and *S6K1* in the G1 and G2 groups were significantly lower (*p* < 0.001). However, there was no distinction between G2 and G0 in terms of the relative expression of mTOR (*p* = 0.122), as shown in Figure 1.

## 4. Discussion

In this study, substituting FSBM for a portion of the FM significantly improved the growth performance in juvenile coho salmon, while substituting unfermented SBM for a portion of the FM significantly decreased these outcomes. The findings demonstrated that some of the FM in the diet for young coho salmon could be replaced by FSBM and that this substitution impact was superior to that of unfermented SBM. The reasons are supposed to be: first, due to the poor palatability of SBM protein, led to a decrease in feed intake, which led to a decrease in essential amino acid intake. The imbalance of amino acid nutrition could lead to decreased growth performance [33]. Second, the antinutrition factors of SBM not only reduced the digestion and absorption of plant protein but also reduced some bioactive factors in feed, thus aggravating the imbalance of amino acids and hindering the growth performance of fish [34, 35]. In addition, the anti-nutrition factors, as a class of highly allergenic proteins, might cause fish body allergy, damage intestinal mucosa, and reduce growth performance [36]. Third, when SBM was fermented, the amount of antinutrition factors was greatly reduced, the amount of crude protein was increased, the amount of small molecular peptides and free amino acids were also enhanced to some extent, and the nutritional value was optimized [37, 38]. According to research by Li et al. [30] and Wang et al. [39], *Aspergillus awamori* and *Bacillus subtilis* dramatically decreased antinutritional components such as raffinose, trypsin inhibitor, glycine, and glycine in SBM while greatly increasing the palatability and nutrient utilization efficiency of SBM. Fourth, this study used a highly efficient protease-producing strain of *B. pumilus*, which could decompose macromolecular proteins into more easily absorbed free amino acids and polypeptide segments. Studies had found that bacteria could metabolize carbohydrates in feed, promote the synthesis of macromolecular proteins, and provide support for fish growth and development [39]. El-haroun et al. [40] showed that *Bacillus* spp. had the effect of decomposing potentially harmful components in feed, producing a large number of essential B vitamins such as vitamin B12 and biotin, and improving food utilization and digestibility.

In this study, substituting FSBM for a portion of the FM significantly improved the activity of the digestive enzymes in juvenile coho salmon, while substituting unfermented SBM for a portion of the FM significantly decreased these outcomes. The reasons are supposed to be: first, soy meal in feed can damage intestinal epithelial cells, interfere with the function of digestive enzymes, and impair intestinal absorption [41]. In addition, the lack of bioactive peptides related to digestion was also an important reason for affecting growth and digestion [14]. Second, the antinutrition factors of SBM could reduce the activities of trypsin, amylase, pepsin, lipase, and other digestive enzymes [42]. For example, trypsin inhibitors could bind to trypsin to inactivate it [43], and phytic acid could bind to the basic protein residues of trypsin and pepsin to reduce enzyme activity [5]. Digestion and nutrient absorption by aquatic animals were mostly carried out by digestive enzymes, and the activities of the enzymes directly reflected the body's capacity for digestion [14]. In this study, FSBM could enhance the activity of digestive enzymes; one of the reasons might be the destruction of antinutrition factors. Similar results were found that feeding FSBM to Atlantic salmon may boost health and growth physiology in fish by promoting intestinal lactic acid bacteria growth, having a prebiotic-like effect, and promoting proximal intestine health by increasing mucin production [44].

The protein content of the animal body represents the degree of absorption and utilization of feed protein to a certain extent [34]. The study found that substituting FSBM for FM dramatically boosted the muscle protein content of juvenile coho salmon but had no discernible impact on crude lipid and ash levels. This was in line with observations in largemouth bass [45] and rainbow trout (*O. mykiss*) [34]. The reasons are supposed to be due to the increase of bioactive peptide content and protease activity during the fermentation process, and the free amino acids and small molecular peptides in the feed increased, which enhanced the digestion and absorption of protein and amino acids, thus facilitating the deposition of protein in juvenile salmon [46]. However, previous studies had shown that there was no significant effect on the muscle compositions of largemouth bass [45] fed the diets which substituted FSBM for a portion of the FM. In addition, Japanese seabass (*Lateolabrax japonicus*) [46] and Giant grouper (*Epinephelus lanceolatus*) [47] fed the diets including a portion of FSBM in place of FM could decrease crude protein and crude lipid contents, and increase the content of moisture. The particular causes of the above inconsistencies may be connected to the type and size of aquatic animals, fermentation strains, feed mix, and other factors, and the specific reasons need further study.

In fish nutrition research, serum biochemical indicators are gradually used as one of the criteria for measuring the health and organ function of aquatic animals and which are influenced by feed ingredients [48, 49]. In fish, serum TP and ALB can indicate protein metabolism and synthesis, as well as amino acid utilization efficiency [50]. The content of serum GLU and T-CHO can be used as an indicator of the body's utilization of nutrients and an important reference index for GLU and lipid metabolism [50, 51]. AKP, as an important part of the detoxification system, is considered to be a potential indicator of stress response and is clinically used for disease diagnosis [52, 53]. According to the study's findings, substituting FSBM for a portion of FM could boost the blood protein level, metabolic ability, and immunological activity of juvenile coho salmon. A high-quality protein substitute for an FM could be an FSBM. The reasons might be that after fermentation by probiotics, the antigen components such as *β*-conglycinin subunits and soybean globulin peptides in SBM were partially or completely decomposed, and then FSBM may boost the physiological functions of fish liver and intestine, and enhance the absorption and utilization of nutrients like GLU, protein, and lipid [54]. Similarly, T-CHO, AKP, and GLU contents in the serum of rainbow trout [40] and juvenile turbot (*Scophthalmus maximus* L.) [55] could be increased by substituting FSBM for a portion of FM.

Excessive oxygen free radicals can cause oxidative damage, and lipid peroxidation is the main problem caused by the imbalance of the antioxidant system [14]. The antioxidant system depends on T-AOC, SOD, and CAT enzyme activity, and MDA level is a key indicator of potential antioxidant capability and the ultimate product of oxidative damage [56]. TNF-*α*, IL-1*β* as well as IL-6, are the proinflammatory cytokines in innate immunity, and the upregulation of proinflammatory cytokines indicates the occurrence of inflammation [57]. In this study, while substituting unfermented SBM for a portion of FM could significantly reduce the activities of antioxidant enzymes and improve the relative expressions of proinflammatory genes in juvenile coho salmon, substituting FSBM for a portion of FM could significantly increase the activities of antioxidant enzymes and improve the relative expressions of proinflammatory genes. The findings demonstrated the coho salmon's immune system and antioxidant capacity could both be improved by FSBM. The reasons are supposed to be: first, Fish may experience oxidative stress due to antinutritional components in plant protein sources, which could be substantially decreased by microbial fermentation of SBM, increasing antioxidant capacity [58]. Studies had shown that probiotic fermentation could remove *β*-conglycinin, soya-saponin, and soybean agglutinin that affect intestinal health or cause inflammatory response [10, 59]. Second, the lipophilic aglycone released from isoflavone glycosides in FSBM was an effective free radical scavenger, and fermentation could effectively improve the bioavailability of isoflavones [60]. In addition, polyphenols were the main natural antioxidants in legumes [39]. The release of bound phenols by microorganisms during fermentation increased the total phenol content, thereby enhancing antioxidant activity [61]. Third, free amino acids such as tyrosine, methionine, histidine, tryptophan, and lysine were generally considered antioxidants, and FSBM could improve antioxidant capacity by increasing the content of free amino acids [62]. In addition, fermentation improved antioxidant activity by increasing the bioavailability of vitamins and extracellular polysaccharides [63]. Fourth, the fermentation process could promote the release of bioactive peptides with immunomodulatory effects in plant proteins [64]. In addition, the bacteria itself contains *β*-glucan, mannose, and other oligosaccharides that may have immune stimulation [65]. Similar studies have shown that Lee et al. [47] used SBM fermented with *Bacillus subtilis* increased the activities of SOD and CAT of rockfish. SBM fermentation with *Lactobacillus plantarum* instead of FM could effectively improve the activity of T-AOC of juvenile turbot [66]. Replacing a FM with FSBM reduced the expression of pro-inflammatory factors *IL-1β*, *IL-12*, *IL-32*, and *TNF-α* in the intestinal tissue of juvenile pearl gentian grouper (*Epinephelus fuscoguttatus♀ × E. lanceolatus♂*) [67]. Compared with unfermented SBM, the expressions of intestinal proinflammatory factors *IL-1β*, *IL-8*, and *TNF-α* were significantly decreased in turbot fed with *Enterococcus faecium* FSBM [68].

The mTOR could respond to extracellular growth factors, amino acids, nutrients, insulin, and other stimuli [69]. The activation of mTOR induced by these stimuli was mediated by PI3K [70]. AKT was a downstream effector of PI3K, which recruited and activated the cell membrane and indirectly activated the mTOR complex [71]. mTOR regulated the phosphorylation and activation of the downstream target ribosomal protein S6 kinases (S6Ks) [72]. The body regulated various physiological activities such as protein synthesis, cell growth, proliferation, and cell cycle through the PI3K-AKT-mTOR signaling pathway. The positive regulation of the mTOR pathway by nutrients was characterized by increased expression of *AKT* and *S6Ks* [73]. In this study, substituting FSBM for a portion of the FM significantly increased the expression of the genes *mTOR*, *AKT*, *PI3K*, and *S6K1* in the liver of juvenile coho salmon, while substituting FSBM for a portion of the FM significantly decreased the expression of the genes *mTOR*, *AKT*, *PI3K*, and *S6K1* in the liver of juvenile coho salmon. The results showed that FSBM could significantly increase the protein synthesis in the liver of juvenile coho salmon, which was consistent with the higher protein content and higher protease activity in the muscle and serum of the G3 group. The reasons are supposed to be: first, fermentation improved the absorption and utilization of nutrients such as protein in SBM. Higher protein and amino acid content in feed could activate the mTOR signaling system [73]. Second, the intake of protein could lead to increase insulin levels in the blood and insulin-induced PI3K-AKT-mTOR signaling pathway gene expression to promote protein synthesis [74]. Studies on rainbow trout had shown that the plant protein sources to replace FM would reduce the expressions of mTOR pathway-related genes and cause growth inhibition, while the FSBM to replace FM would positively regulate mTOR pathway-related genes [74]. The increase in protein synthesis is beneficial to protein deposition, which is the basis for the growth and development of aquatic animals [75].

## 5. Conclusion

In summary, the SBM protein fermented by *B. pumilus* to replace 10% FM protein had a positive effect on the growth performance, protein deposition, antioxidant enzyme activity, digestive enzyme activity, protein synthesis, and immune-related genes of juvenile coho salmon. This study provides a support for the application of FSBM in coho salmon feed.

## Figures and Tables

**Table 1:** Experimental diet formula (g/100 g of dried feed) and proximate composition (%, dry matter percentage).

Ingredients	G0	G1	G2	G3
Fish meal	40.07	25.99	25.99	25.99
Soybean meal	0.00	21.20	10.76	0.00
Fermented soybean meal	0.00	0.00	7.95	15.89
Chicken meat powder	10.02	10.00	10.00	10.00
Shrimp meal	10.02	10.00	10.00	10.00
High gluten flour	17.88	17.84	17.84	17.84
*α*-Starch	3.01	3.01	3.01	3.01
*α*-Cellulose	8.53	0.00	2.49	5.31
Fish oil	4.01	5.51	5.51	5.51
Soybean oil	4.01	4.00	4.00	4.00
Ca(H_2_PO_4_)_2_	1.01	1.01	1.01	1.01
Mineral premix^1^	0.52	0.52	0.52	0.52
Vitamin premix^2^	0.52	0.52	0.52	0.52
Choline chloride	0.30	0.30	0.30	0.30
Vitamin C	0.10	0.10	0.10	0.10
Approximate composition				
Crude protein (%)	42.68	42.45	42.34	42.36
Crude lipid (%)	15.21	15.22	15.21	15.20
Ash (%)	7.26	7.64	7.54	7.33
Moisture (%)	9.52	9.57	9.53	9.49
Crude fiber (%)	3.35	4.60	4.41	4.20
Nitrogen-free extract (%)	21.98	20.52	20.97	21.42
Gross energy (MJ/kg)	21.43	21.15	21.27	21.34

*Note*: ^1^Composition (mg/kg mineral premix): AlK(SO_4_)_2_·12H_2_O, 123.7; CaCl_2_, 17,879.8; CuSO_4_·5H_2_O, 31.7; CoCl_2_·6H_2_O, 48.9; FeSO_4_·7H_2_O, 707.4; MgSO_4_·7H_2_O, 4316.8; MnSO_4_·4H_2_O, 31.1; ZnSO_4_·7H_2_O, 176.7; KCl, 1191.9; KI, 5.3; NaCl, 4934.5; Na_2_SeO_3_·H_2_O, 3.4; Ca(H_2_PO_4_)_2_·H_2_O, 12457.0; KH_2_PO_4_, 9930.2. ^2^Composition (IU or g/kg vitamin premix): retinal palmitate, 10,000 IU; cholecalciferol, 4000 IU; *α*-tocopherol, 75.0 IU; menadione, 22.0 g; thiamine HCl, 40.0 g; riboflavin, 30.0 g; D-calcium pantothenate, 150.0 g; pyridoxine HCl, 20.0 g; meso-inositol, 500.0 g; D-biotin, 1.0 g; folic acid, 15.0 g; ascorbic acid, 200.0 g; niacin, 300.0 g; cyanocobalamin, 0.3 g. All the feed materials above are provided by Shandong Conqueren Marine Technology Co., Ltd., Weifang, China. Fish meal (Anchovy, *Engraulis japonicus*): protein content 69.88%, lipid content 8.00%. Soybean meal: protein content 46.81%, lipid content 1.84%. Fermented soybean meal: protein content 55.21%, lipid content 1.93%. Chicken powder: protein content 62.00%, lipid content 12.00%. Shrimp powder: protein content 49.00%, lipid content 8.00%. High gluten flour: protein content 11.00%, lipid content 1.60%. Fish oil: Pacific herring, *Clupea pallasi*; Mackerel, *Pneumatophorus japonicus*.

**Table 2:** Nutritional composition and antinutritional factor content of soybean meal and fermented soybean meal.

Index	Soybean meal	Fermented soybean meal
Crude protein (%)	46.81 ± 0.21	55.21 ± 0.33
Crude lipid (%)	1.84 ± 0.05	1.93 ± 0.09
Crude fiber (%)	8.84 ± 0.06	6.13 ± 0.11
Crude ash (%)	8.62 ± 0.22	7.45 ± 0.19
Moisture (%)	9.35 ± 0.21	9.64 ± 0.15
Nitrogen-free extract (%)	24.54 ± 0.14	19.64 ± 0.17
Gross energy (kJ/kg)	3,373.20 ± 21.58	3,412.90 ± 22.36
Polypeptide (%)	1.39 ± 0.06	21.83 ± 0.21
Trypsin inhibitors (mg/g)	66.13 ± 1.58	11.35 ± 0.95
Glycinin (mg/g)	141.13 ± 3.65	24.15 ± 1.75
*β*-Conglycinin (mg/g)	105.01 ± 2.74	26.39 ± 1.16
Urease (U/g)	8.01 ± 0.18	1.09 ± 0.05
pH	7.15 ± 0.02	6.44 ± 0.04

**Table 3:** Forward and reverse primers of the genes for real-time quantitative PCR.

Gene	Primer sequence	Genbank	Amplicon size (bp)	TM (°C)
*β-Actin* ^1^	F: CCAAAGCCAACAGGGAGAA R: AGGGACAACACCGCCTGGAT	XM_031822094.1	91	60
*PI3K* ^2^	F: CCAGTGGCTCAAGGACAAGAACAG R: GGATGAAGGTGGCTACGCAGTATC	XM_020466892.2	98	60
*AKT* ^3^	F: GCAGCCATCCTACAAATC R: TGAAACAGGGTCCACAAG	XM_031814748.1	178	60
*mTOR* ^4^	F: CTTCGCCAACTACCTCCG R: TGCCCTCTTCACCTCAAACT	XM_020506200.2	139	60
*S6K1* ^5^	F: CAGCACCTGAGCAGCAGTTAGC R: CTCGGATCGGCAGTGGAAAGTTC	XM_020465833.2	131	60
*IL-1β* ^6^	F: GCGACATGGTGCGTTTCCTTTT R: TGTCTACCGGTTTGGTGTAGTCCT	XM_020475860.2	129	60
*IL-6* ^7^	F: GAGCTACGTAACTTCCTGGTTGAC R: GCAAGTTTCTACTCCAGGCCTGAT	XM_020507339.2	134	60
*TNF-α* ^8^	F: GGCGAGCATACCACTCCTCT R: TCGGACTCAGCATCACCGTA	XM_020497470.2	125	60

*Note*: ^1^*β-Actin*, reference gene. ^2^*PI3K*, phosphatidylinositide 3-kinases. ^3^*AKT*, serine/threonine kinase, or protein kinase B (PKB). ^4^*mTOR*, mechanistic target of rapamycin. ^5^*S6K1*, ribosomal protein S6 kinase 1. ^6^*IL-1β*, interleukin 1*β*. ^7^*IL-6*, interleukin 6. ^8^*TNF-α*, tumor necrosis factor *α*.

**Table 4:** Effects of replacing fish meal with fermented or/and unfermented soybean meal on survival and growth performance of juvenile coho salmon.

Index	G0	G1	G2	G3	*p*-Value
Initial weight (g)	152.23 ± 3.21	152.23 ± 3.21	152.23 ± 3.21	152.23 ± 3.21	1.000
Final weight (g)	570.01 ± 10.15^b^	458.33 ± 1.15^d^	529.33 ± 7.37^c^	602.05 ± 3.01^a^	<0.001
SR^1^ (%)	93.33 ± 1.92	91.11 ± 2.94	92.22 ± 2.22	94.44 ± 1.11	0.728
WGR^2^ (%)	274.44 ± 6.95^b^	201.08 ± 3.46^d^	247.72 ± 4.65^c^	295.49 ± 4.62^a^	<0.001
SGR^3^ (%/day)	1.57 ± 0.03^b^	1.31 ± 0.01^d^	1.48 ± 0.02^c^	1.64 ± 0.02^a^	<0.010
HSI^4^ (%)	1.56 ± 0.02^c^	1.68 ± 0.01^a^	1.64 ± 0.02^b^	1.51 ± 0.04^c^	0.332
VSI^5^ (%)	10.45 ± 0.35^b^	11.60 ± 0.38^a^	11.57 ± 0.36^a^	9.64 ± 0.41^c^	<0.012
FCR^6^ (%)	1.79 ± 0.04^c^	2.36 ± 0.01^a^	1.98 ± 0.02^b^	1.66 ± 0.02^d^	<0.011
CF^7^ (g/cm^3^)	1.85 ± 0.04^b^	1.57 ± 0.05^d^	1.68 ± 0.03^c^	1.99 ± 0.06^a^	<0.011
FI (g)	751.77 ± 1.51	751.31 ± 2.09	751.78 ± 1.38	750.71 ± 2.44	0.974

*Notes*: G0 is the control group. G1 is the SBM group. G2 is the SBM and FSBM group. G3 is the FSBM group. All above data are mean ± SD (*n* = 3 × 3), which means three parallel groups for each experimental group, and three fish samples for each parallel group, except SR is mean ± SD (*n* = 3 × 30), which means three parallel groups for each experimental group and 30 fish samples for each parallel group. Different superscript letters in the same row indicate significant differences among the data (*p* < 0.05). ^1^SR, survival rate. ^2^WGR, weight growth rate. ^3^SGR, specific growth rate. ^4^HSI, hepatosomatic index. ^5^VSI, viscerosomatic index. ^6^FCR, feed coefficient ratio. ^7^CF, condition factor. ^8^FI, food intake.

**Table 5:** Effects of replacing fish meal with fermented or/and unfermented soybean meal on the moisture, crude protein, crude lipid, and ash in the muscle of juvenile coho salmon (%/g of wet weight).

Index	G0	G1	G2	G3	*p*-Value
Moisture (%)	75.18 ± 0.29^a^	75.32 ± 0.36^a^	75.54 ± 0.61^a^	74.28 ± 0.43^b^	0.288
Crude protein (%)	18.26 ± 0.27^b^	17.50 ± 0.18^c^	18.09 ± 0.25^b^	19.00 ± 0.21^a^	0.007
Crude lipid (%)	4.94 ± 0.11	5.01 ± 0.13	4.99 ± 0.19	4.97 ± 0.15	0.959
Ash (%)	1.47 ± 0.22	1.58 ± 0.13	1.40 ± 0.08	1.39 ± 0.14	0.532

*Notes*: G0 is the control group. G1 is the SBM group. G2 is the SBM and FSBM group. G3 is the FSBM group. All above data are mean ± SD (*n* = 3 × 3), which means three parallel groups for each experimental group and three fish samples for each parallel group. Different superscript letters in the same row indicate significant differences among the data (*p* < 0.05).

**Table 6:** Effects of replacing fish meal with fermented or/and unfermented soybean meal on serum biochemical indexes of juvenile coho salmon.

Index	G0	G1	G2	G3	*p*-Value
GLU^1^ (mmol/L)	5.81 ± 0.15^b^	4.13 ± 0.06^d^	4.77 ± 0.14^c^	6.16 ± 0.09^a^	<0.001
T-CHO^2^ (mmol/L)	4.71 ± 0.15^b^	3.62 ± 0.11^d^	4.32 ± 0.14^c^	5.10 ± 0.12^a^	<0.001
TP^3^ (g/L)	57.54 ± 1.12^b^	40.07 ± 1.37^d^	45.04 ± 2.11^c^	63.20 ± 1.35^a^	<0.001
ALB^4^ (g/L)	27.82 ± 1.93^b^	18.82 ± 0.95^d^	23.25 ± 1.36^c^	33.08 ± 1.86^a^	<0.001
AKP^5^ (U/mL)	19.81 ± 1.55^b^	12.79 ± 0.75^d^	16.07 ± 0.83^c^	24.30 ± 1.31^a^	<0.001

*Notes*: G0 is the control group. G1 is the SBM group. G2 is the SBM and FSBM group. G3 is the FSBM group. All above data are mean ± SD (*n* = 3 × 3), which means three parallel groups for each experimental group and three fish samples for each parallel group. Different superscript letters in the same row indicate significant differences among the data (*p* < 0.05). ^1^GLU, glucose. ^2^T-CHO, total cholesterol. ^3^TP, total protein. ^4^ALB, albumin. ^5^AKP, alkaline phosphatase.

**Table 7:** Effects of replacing fish meal with fermented or/and unfermented soybean meal on the activity of digestive enzyme in the stomach and intestine of juvenile coho salmon.

Index	G0	G1	G2	G3	*p*-Value
Pepsin (U/mg)	15.48 ± 0.45^b^	8.18 ± 0.23^d^	12.23 ± 0.38^c^	16.95 ± 0.37^a^	<0.001
Trypsin (U/mg)	2,368.31 ± 130.02^b^	1,569.23 ± 124.41^d^	2,122.64 ± 88.39^c^	2,678.84 ± 112.26^a^	<0.001
*α*-Amylase (U/mg)	1.43 ± 0.03^b^	0.84 ± 0.01^d^	1.24 ± 0.05^c^	1.52 ± 0.04^a^	<0.001
Lipase (U/mg)	30.18 ± 1.16^b^	24.71 ± 0.79^d^	27.55 ± 0.82^c^	33.57 ± 0.94^a^	<0.001

*Notes*: G0 is the control group. G1 is the SBM group. G2 is the SBM and FSBM group. G3 is the FSBM group. All above data are mean ± SD (*n* = 3 × 3), which means three parallel groups for each experimental group and three fish samples for each parallel group. Different superscript letters in the same row indicate significant differences among the data (*p* < 0.05).

**Table 8:** Effects of replacing fish meal with fermented or/and unfermented soybean meal on antioxidant capacity in the liver of juvenile coho salmon.

Index	G0	G1	G2	G3	*p*-Value
T-AOC^1^ (mmol/g)	2.46 ± 0.11^b^	1.56 ± 0.05^d^	1.96 ± 0.08^c^	2.65 ± 0.07^a^	<0.001
CAT^2^ (U/mg)	322.22 ± 17.34^b^	220.46 ± 12.47^d^	268.95 ± 22.43^c^	364.45 ± 13.85^a^	<0.001
SOD^3^ (U/mg)	827.56 ± 31.27^b^	493.26 ± 16.43^d^	692.36 ± 34.45^c^	890.25 ± 21.30^a^	<0.001
MDA^4^ (nmol/mg)	3.19 ± 0.20^c^	4.29 ± 0.14^a^	3.76 ± 0.05^b^	2.54 ± 0.12^d^	<0.001

*Notes*: G0 is the control group. G1 is the SBM group. G2 is the SBM and FSBM group. G3 is the FSBM group. All above data are mean ± SD (*n* = 3 × 3), which means three parallel groups for each experimental group and three fish samples for each parallel group. Different superscript letters in the same row indicate significant differences among the data (*p* < 0.05). ^1^T-AOC, total antioxidant capacity. ^2^CAT, catalase. ^3^SOD, superoxide dismutase. ^4^MDA, malondialdehyde.

## Data Availability

The data that support the findings of this study are available from the corresponding author upon reasonable request.
